# Injectable cartilage microtissues based on 3D culture using porous gelatin microcarriers for cartilage defect treatment

**DOI:** 10.1093/rb/rbae064

**Published:** 2024-06-04

**Authors:** Jing Zhu, Qiuchen Luo, Tiefeng Cao, Guang Yang, Lin Xiao

**Affiliations:** School of Biomedical Engineering, Shenzhen Campus of Sun Yat-Sen University, Shenzhen 518107, China; School of Biomedical Engineering, Shenzhen Campus of Sun Yat-Sen University, Shenzhen 518107, China; Department of Gynaecology, First Affiliated Hospital of Sun Yat­Sen University, Guangzhou 510070, China; Department of Biomedical Engineering, College of Life Science and Technology, Huazhong University of Science and Technology, Wuhan 430074, China; School of Biomedical Engineering, Shenzhen Campus of Sun Yat-Sen University, Shenzhen 518107, China

**Keywords:** cartilage repair, microtissue engineering, microcarriers, gelatin, BMSCs

## Abstract

Cartilage tissues possess an extremely limited capacity for self-repair, and current clinical surgical approaches for treating articular cartilage defects can only provide short-term relief. Despite significant advances in the field of cartilage tissue engineering, avoiding secondary damage caused by invasive surgical procedures remains a challenge. In this study, injectable cartilage microtissues were developed through 3D culture of rat bone marrow mesenchymal stem cells (BMSCs) within porous gelatin microcarriers (GMs) and induced differentiation. These microtissues were then injected for the purpose of treating cartilage defects *in vivo*, via a minimally invasive approach. GMs were found to be noncytotoxic and favorable for cell attachment, proliferation and migration evaluated with BMSCs. Moreover, cartilage microtissues with a considerable number of cells and abundant extracellular matrix components were obtained from BMSC-laden GMs after induction differentiation culture for 28 days. Notably, ATDC5 cells were complementally tested to verify that the GMs were conducive to cell attachment, proliferation, migration and chondrogenic differentiation. The microtissues obtained from BMSC-laden GMs were then injected into articular cartilage defect areas in rats and achieved superior performance in alleviating inflammation and repairing cartilage. These findings suggest that the use of injectable cartilage microtissues in this study may hold promise for enhancing the long-term outcomes of cartilage defect treatments while minimizing the risk of secondary damage associated with traditional surgical techniques.

## Graphical abstract

**Figure:**
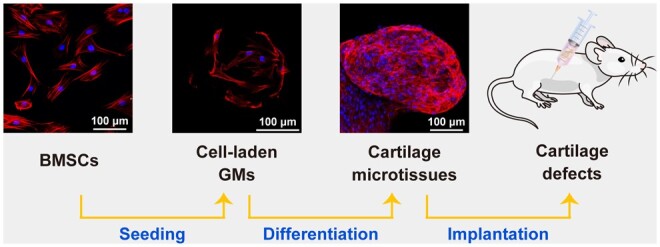

## Introduction

Cartilage tissue is devoid of blood vessels, nerves and lymph, which contributes to its limited self-repair ability [1, 2]. Therefore, articular cartilage lesions do not heal spontaneously, and irreversible degenerative processes ensue [3], including microdamage to the extracellular matrix (ECM), chondrocyte abnormalities and deaths, progressive degeneration of the synovial joint, cartilage defects and subsequent subchondral bone defects, which are inexorably accompanied by the development of osteoarthritis (OA) [4, 5]. Currently, clinical treatment strategies for articular cartilage defects mainly include osteochondral autograft transfer, osteochondral allograft transplantation, microfracture and autologous chondrocyte implantation (ACI) [6–8]. They are selected by the surgeon based on patient-specific factors such as age, activity level and the condition of the defect. However, current surgical approaches have limited ability for tissue regeneration and only provide short-term relief, which is insufficient for preventing the development and progression of OA [9].

Fortunately, tissue engineering approaches are promising alternatives providing long-term solutions for cartilage repair [10, 11]. For example, Nie *et al.* [12] cocultured allogeneic porcine chondrocytes and alginate hydrogels for 35 days to form cartilage neotissue. They developed a decellularized allogeneic hyaline cartilage graft whose therapeutic efficacy in articular cartilage defects surpassed that of ACI after implantation in porcine knee joints. However, the implantation of bulk scaffolds or engineered tissues often requires invasive surgical operations, which may cause secondary damage and increase the risk of inflammation, among other side effects [13, 14]. In contrast, the microtissue strategy shows great application prospects in an injectable manner with minimal invasiveness, fitting defects with complex shapes and efficiently promoting tissue regeneration [15–17]. Simultaneously, microtissues can be precisely designed and manufactured to achieve a biomimetic cell density and microenvironment, establish effective substance exchange and cell connections and facilitate extracellular matrix deposition [18–21].

Microtissue approaches have received increasing concerns in the field of articular cartilage repair [22]. For instance, Yin *et al.* [23, 24] prepared natural cartilage ECM-derived particles through pulverization, size screening and decellularization processes; these particles were used to carry stem cells, promote chondrogenic differentiation and form cartilage microtissues to facilitate *in vivo* cartilage regeneration. Compared with solid particles, porous microsphere-based microcarriers (MCs) exhibit interconnected external and internal pores that permit efficient transport of growth factors, nutrients and metabolic waste while providing immense surface area for cell adhesion, proliferation and migration [25, 26]. In addition, their microarchitectural characteristics could facilitate cell–cell and cell–ECM interactions and play a supporting and protective role in reducing cell damage during injection [27]. Recently, Wang *et al.* [28] fabricated ECM-mimicking nanofibrous microcarriers (NF-MCs) by complexing bacterial cellulose, amino acids and chitosan. The structurally embedded biomimetic NF components in the macroporous MCs were used to generate functional cartilage microtissues that healed osteochondral defects.

Gelatin is obtained by the degradation and denaturation of collagen [29, 30], one of the major components of the ECM [31]. Due to its great biocompatibility, biodegradability, negligible immunogenicity and low cost, gelatin has been widely utilized for tissue engineering [13, 32]. Moreover, a sufficient peptide sequence of arginine-glycine-aspartate (RGD) can activate cellular integrin-mediated signaling pathways, permitting cell adhesion and growth and improving the secretion of collagen fibers [33, 34]. 3D TableTrix^®^ gelatin microcarriers (GMs) were first developed by Li *et al.* in 2014 [35]. They have interconnected macroporous structures with porosities of 95.73% ± 0.28% and pore sizes in the range of 30–80 μm, preferably facilitating cell loading and maintaining cell viability and functionality [36]. To date, GMs have been applied as bioreactors for *in vitro* cell expansion [37, 38], exosome collection [39, 40] and the fabrication of engineered meatballs [41–44]. Furthermore, cell-laden GMs have shown promising therapeutic effects *in vivo* in various experimental models, including skin wound healing [13], limb ischemia [45, 46], liver cirrhosis [47], intervertebral discs [48] and OA [49].

**Figure 1.**
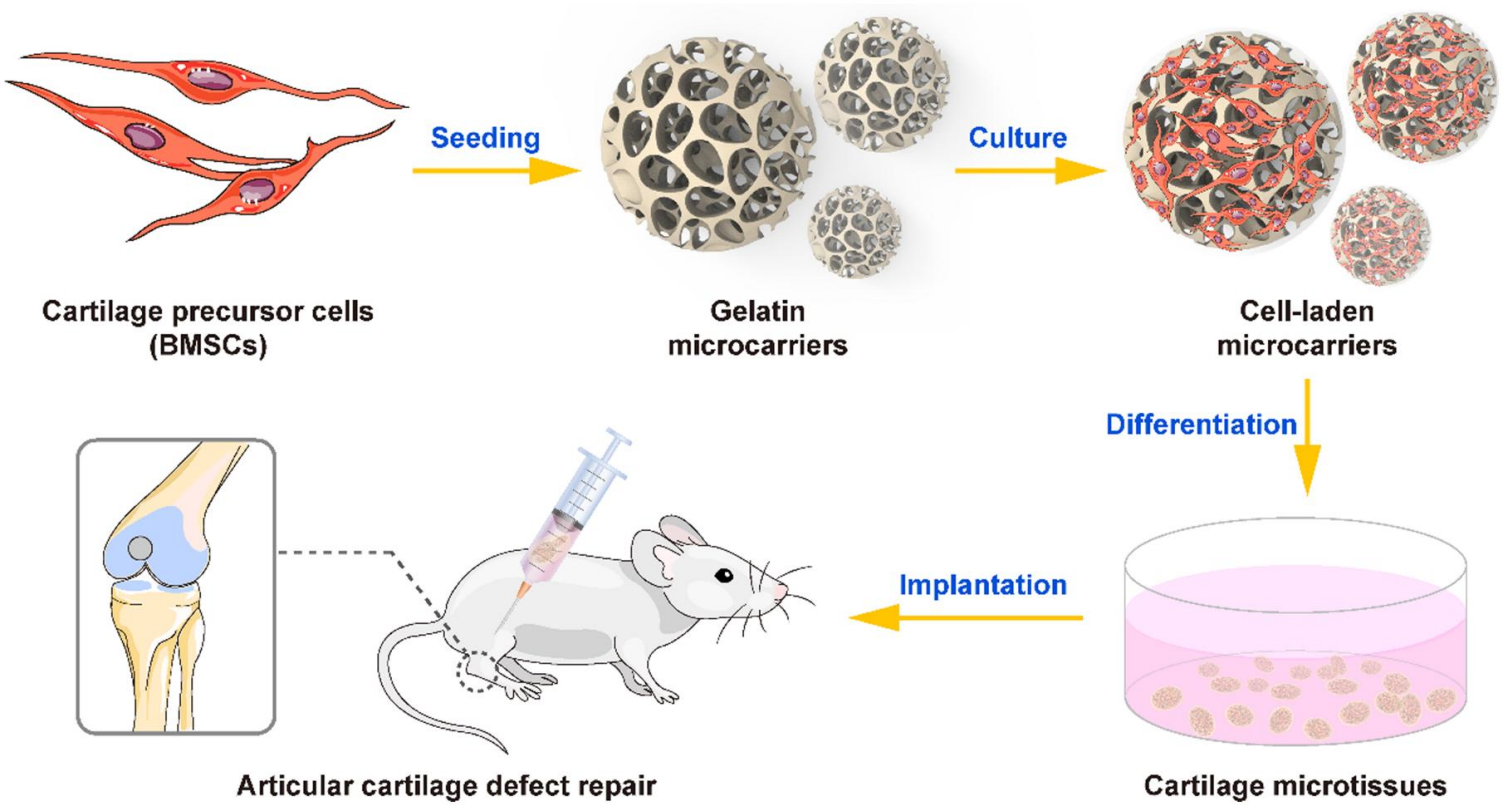
Schematic diagram of the construction of cartilage microtissues based on GMs and their application in the repair of articular cartilage defects.

In this work, GMs were employed to construct cartilage microtissues that were subsequently used to treat cartilage defects in an injectable manner (Figure 1). Bone marrow mesenchymal stem cells (BMSCs), characterized by their easy accessibility, self-renewal capacity, low immunogenicity and safety [50], were used as the seed cells in this study. BMSCs were seeded into GMs to obtain cell-laden GMs, which were then induced to differentiate and form cartilage microtissues (Figure 1). These microtissues were injected to treat articular cartilage defects *in vivo* in a minimally invasive manner. Notably, to verify that the GMs were conducive to cell attachment, proliferation, migration and chondrogenic differentiation, ATDC5, a mouse cartilage precursor cell line derived from teratoma cells, was complementally utilized and tested for cell viability, proliferation and chondrogenic differentiation on GMs. ATDC5 has been widely used to explore biochemical and developmental mechanisms throughout the chondrogenic differentiation process [51–53]. However, due to the abnormal cellular phenotype and potential carcinogenic risk of ATDC5, ATDC5-laden GMs were not evaluated at the tissue level or *in vivo* in this study.

## Materials and methods

### Materials

Dulbecco’s modified Eagle’s medium/Ham’s F-12 (DMEM/F-12), antibiotics (100 mg/ml streptomycin and 100 U/ml penicillin), 0.25% trypsin, PBS buffer, ITS-A, ELISA kits, FGF-basic, TGF-β1, and fetal bovine serum (FBS) were obtained from Thermo Fisher Scientific (China) Co., Ltd (Shanghai, China). Dexamethasone, L-proline, and L-ascorbic acid were purchased from Sigma-Aldrich Trading Co., Ltd (Shanghai, China). Cell Counting Kit-8 (CCK-8) reagent was supplied by Langeke Technology Co., Ltd (China). 3D FloTrix^®^ Digest reagent and GMs (3D TableTrix^®^) were supplied by Beijing Huayu Technology Co., Ltd (Beijing, China). Calcein-AM/PI double staining kit, TRITC phalloidin and 4′,6-diamidino-2-phenylindole (DAPI) were obtained from Yongqinquan Intelligent Equipment Co., Ltd (Suzhou, China). The PrimeScript™ RT reagent kit and TB Green Premix Ex Taq II reagent kit were purchased from Takara Biomedical Technology Co., Ltd (Beijing, China). H&E, SO/FG, AB, toluidine blue and ethylenediaminetetraacetic acid (EDTA) were obtained from Wuhan Servicebio Technology Co., Ltd (Wuhan, China). All chemicals were of analytical grade and were used without further purification.

### Cells and animals

BMSCs were isolated from the bone marrow cavities of the femurs and tibias of Sprague Dawley (SD) rat (4 weeks old, body weight: 80–180 g). ATDC5 cells were obtained from the European Collection of Authenticated Cell Cultures (ECACC). Both BMSCs and ATDC5 cells were cultured in complete growth medium (89% DMEM/F-12 supplemented with 10% FBS and 1% antibiotics) in an incubator at 37°C with 5% CO_2_. The medium was changed every 2 days, and the cells were passaged before reaching 80% confluence. BMSCs were used until passage four (P4).

SD rats were provided by the Institute of Biological and Medical Engineering, Guangdong Academy of Sciences (Guangzhou, China). All animal studies were performed according to the research protocol approved by the Animal Experimentation Ethics Committee of the Institute of Biological and Medical Engineering, Guangdong Academy of Sciences (license No. of laboratory animal facility: SYXK (Guangdong) 2018-0183).

### Characterization of GMs

The morphology of the GMs was monitored by scanning electron microscopy (SEM) at an accelerating voltage of 20.0 kV. A Fourier transform infrared (FTIR) spectrophotometer was used to characterize the chemical composition of the GMs. Spectral measurements were performed within the mid-infrared range of 4000–500 cm^−1^. All measurements were performed in a dry atmosphere at room temperature, and the background spectrum was obtained from a pure KBr pellet.

### Cytotoxicity assay of GMs

The cytotoxicity of GMs was tested in a noncontact manner by a CCK-8 assay. Briefly, different weights of GMs were immersed in the culture medium for 3 days at concentrations of 4, 2 and 1 mg/mL. The extract solutions were collected by centrifuging at 1000 rpm for 5 min. In addition, BMSCs in the logarithmic growth phase were seeded in 96-well plates at a density of 5 × 10^3^/well and cultured for 24 h. Then, the culture medium was replaced with the extract solutions, and the BMSCs were subsequently cultured for 24, 48 and 72 h. Afterwards, cell viability was tested and calculated by a CCK-8 assay according to the manufacturer’s instructions.

### 3D cell culture on GMs

3D TableTrix^®^ Microcarrier Series (2.5 × 10^4^ GMs per piece) were placed into nontissue culture-treated 6-well plates. Cell (BMSC or ATDC5) suspensions (500 µl) were uniformly dropped onto a MC series at densities of 6.25 × 10^4^, 1.25 × 10^5^, 3.125 × 10^5^, 6.25 × 10^5^ and 1.25 × 10^6^ cells/mL, in which the cell-to-GM ratios were 5:1, 10:1, 25:1, 50:1 and 100:1, respectively. PBS was added to the well gaps of the plates to prevent rapid evaporation of the culture medium. After incubation at 37°C for 2 h, each well was supplemented with 6 mL of medium, which was subsequently refreshed every 2 days.

### Cell activity assay

Cell activity after 3D culture on GMs for 1, 3, 5 and 7 days was observed using a calcein-AM/propidium iodide (PI) double staining kit. Specifically, cell-laden GMs were gently washed with PBS to remove the original medium. Then, they were immersed in PI solution (8 µM) and incubated for 10 min at room temperature, followed by incubation in AM solution (2 µM) for 30 min. After the live and dead cells were stained green and red respectively, they were imaged with a laser-scanning confocal microscope (TCS-SP5, Leica, Germany) with *Z*-axis scans (10 µm per layer). Calcein-AM: green, *λ*_ex_ = 490 nm, *λ*_em_ = 515 nm; PI: red, *λ*_ex_ = 535 nm, *λ*_em_ = 617 nm. To illustrate the relative number of live cells on GMs, the fluorescence intensity of live cells was quantified by ImageJ software and normalized to that at 5:1 on Day 1.

### Morphology characterization of cell-laden GMs by SEM

After washing with PBS, the cell-laden GMs were fixed in 2.5% glutaraldehyde for 2.5–3 h at room temperature, followed by dehydration with a graded ethanol series (30, 50, 70, 90, 95 and 100%) for 10 min in each solution. After air drying, they were carefully placed on conductive adhesive tape and then sputter-coated with gold for SEM observation.

### Chondrogenic differentiation of cell-laden GMs

BMSCs and ATDC5 cells were respectively seeded into GMs at a ratio of 100:1. After incubation in complete culture medium for 24 h, the cell-laden GMs were transferred and cultured in chondrogenic differentiation medium for 28 days, during which the medium was refreshed every 2 days. The chondrogenic differentiation medium of BMSCs consisted of DMEM/F-12 (88%), FBS (10%), antibiotics (1%), insulin-transferrin-selenium-A (ITS-A, 1%, 125 mg/L), FGF-basic (10 ng/mL), TGF-β1 (10 ng/mL), dexamethasone (100 nM), L-proline (40 µg/mL) and L-ascorbic acid (50 µg/mL), while that of ATDC5 cells contained DMEM/F-12 (88% v/v), FBS (10%), antibiotics (1%) and ITS-A (1%, 125 mg/L).

### Quantitative real-time polymerase chain reaction

BMSCs were dissociated from cell-laden GMs with 3D FloTrix^®^ Digest reagent. Afterwards, the mRNA expression levels of aggrecan (*ACAN*), proteoglycan 4 (*PRG4*), collagen type II alpha 1 (*COL2A1*), sex determining region Y and SRY box 9 (*SOX9*), pannexin 1 (*Panx1*) and connexin 43 (*Cx43*) were detected by quantitative real-time polymerase chain reaction (RT-qPCR). ATDC5 cells were also dissociated from GMs, and the mRNA expression of *ACAN*, *PRG4*, *COL2A1* and *SOX9* was detected. Briefly, total cellular RNA was extracted from cells using TRIzol reagent and reverse-transcribed into cDNA with a PrimeScript™ RT reagent kit. RT-qPCR was performed on a StepOne™ system (ABI 7500, US) using a TB Green Premix Ex Taq II reagent kit. Gene expression levels were normalized to the reference gene *GAPDH* (glyceraldehyde-3-phosphate dehydrogenase) and evaluated using the 2^−ΔΔCT^ method. The primers used are listed in Table S1.

### Cell morphological analysis

After chondrogenic differentiation for 0, 14 and 28 days, cell morphology was observed by cytoskeleton staining, where actin was stained red with TRITC phalloidin. Briefly, cell-laden GMs were fixed with formaldehyde solution (4%) for 10–30 min and permeabilized with Triton X-100 (0.5%) for 5 min. Then, TRITC phalloidin (100 nM) was used to cover and incubate the cell-laden GMs at room temperature in the dark for 30 min. The nuclei were counterstained with DAPI solution for 10–30 min. After washing with PBS, the samples were imaged with a laser-scanning confocal microscope (TCS-SP5, Leica, Germany). TRITC: red, *λ*_ex_ = 545 nm, *λ*_em_ = 570 nm; DAPI, blue, *λ*_ex_ = 364 nm, *λ*_em_ = 454 nm.

Additionally, to exclude the effect of gelatin composition on cells, the phenotype of BMSCs cultured in 2D plates supplemented with GM extract was also observed. GM extract was obtained by immersing GMs in cell culture medium at the same concentration as that used for landing BMSCs on GMs. After extraction for 72 h, the extract was collected by centrifugation to remove GMs.

### Histological analysis

Histological staining, including H&E, SO/FG and AB staining, was used to analyze the deposition of ECM and the formation of cartilage microtissues from BMSC-laden GMs. After discarding the original medium, the GMs were washed twice with PBS and fixed in formaldehyde solution (4%) for 1–2 days. The GMs were subsequently embedded in agarose and paraffin, sectioned, and stained following the manufacturer’s instructions.

### Construction of the cartilage defect model and treatment

Fifty-four SD rats (6 weeks old, body weight: 260–280 g) were used to create knee cartilage defect models to evaluate the repair efficiency of the microtissues. The rats were equally and randomly divided into a sham-operated group, a blank control group and an implantation treatment group (*n* = 6 for each group at each time point). Following general anesthesia, a longitudinal skin incision (length: 2 cm) was created on the surface of the right knee joint. The knee joint was opened through a medial parapatellar approach. The patella was dislocated laterally to expose the femoropatellar groove. Cartilage defects (diameter: 3 mm, height: 1.5 mm) were subsequently constructed on the patellar groove by using a 3 mm diameter hand drill, after which cartilage microtissues from BMSC-laden GMs were implanted into the defects. In addition, the cartilage defects of the rats in the blank control group were not treated, while those in the sham-operated group were not constructed. After the surgery, the animals were returned to their cages without joint immobilization.

### Inflammation assessment

At 2, 4 and 8 weeks postsurgery, serum was collected to assess the inflammatory response in the rats. Briefly, rats were anesthetized with an intraperitoneal injection of chloral hydrate (10%, 3.5 mL/kg). After opening the abdominal cavity, the intestine was pushed to one side to fully expose the abdominal aorta, from which 5 ml of blood was collected. Following static settlement and centrifugation (3000 r/min, 15 min), serum was collected, and IL-1β, TNF-α and IL-6 were quantified by ELISA kits according to the manufacturer’s instructions.

### Gross observation and histological analysis

At predetermined time points, after abdominal aorta blood collection, the right knee joints were removed and photographed. Following fixation in formaldehyde solution (4%) for 1–2 days, the joints were immersed in 10% EDTA solution until decalcification was complete (1–1.5 months). Subsequently, the tissues were embedded in paraffin, sectioned and subjected to H&E staining, SO/FG staining, toluidine blue staining and immunohistochemical (IHC) staining for Col2a1 and AGG. For histological grading, sections corresponding to the center of each defect were selected and subjected to blind evaluation by three investigators. The histological grading scale (Table S2) described by Wakitani *et al.* [54, 55] comprises five categories, the sum of which is scored from 0 (normal cartilage) to 14 (no repair tissue).

### Statistical analysis

All data in this study are presented as the means ± standard deviations (SDs). Statistical analysis was performed using SPSS 26.0 software. The statistical significance of differences between two groups was determined by a two-tailed independent-samples *t* test. The statistical significance of differences among multiple groups was determined via one-way analysis of variance (ANOVA) using the Tukey *post hoc* test. The levels of significance were established at * *P *≤* *0.05, ** *P *≤* *0.01 and *** *P *≤* *0.001.

## Results and discussion

### Construction of cell-laden GMs

The 3D TableTrix^®^ GMs used in this study have interconnected macroporous structures with a porosity of 95.73% ± 0.28%, pore sizes in the range of 30–80 μm, and a size distribution ranging from 150 to 300 μm [35, 36]. Their morphology was visualized by SEM (Figure S1A). Their chemical composition was characterized by FTIR (Figure S1B). Five major peaks, i.e. at 3301.4, 1654.8, 1535.9 and 1450.4 cm^−1^, as well as 1229.5 cm^−1^ correspond to the amide A, amide I, amide II and amide III regions of gelatin, respectively [56].

GMs have good biocompatibility and the ability to maintain cell viability [36]. This was verified by a noncontact cytotoxicity assay using BMSCs. The results showed that the cell viability was maintained at over 80% at all the examined material concentrations (1, 2 and 4 mg/mL) for different incubation time (24, 48 and 72 h) (Figure S2).

**Figure 2.**
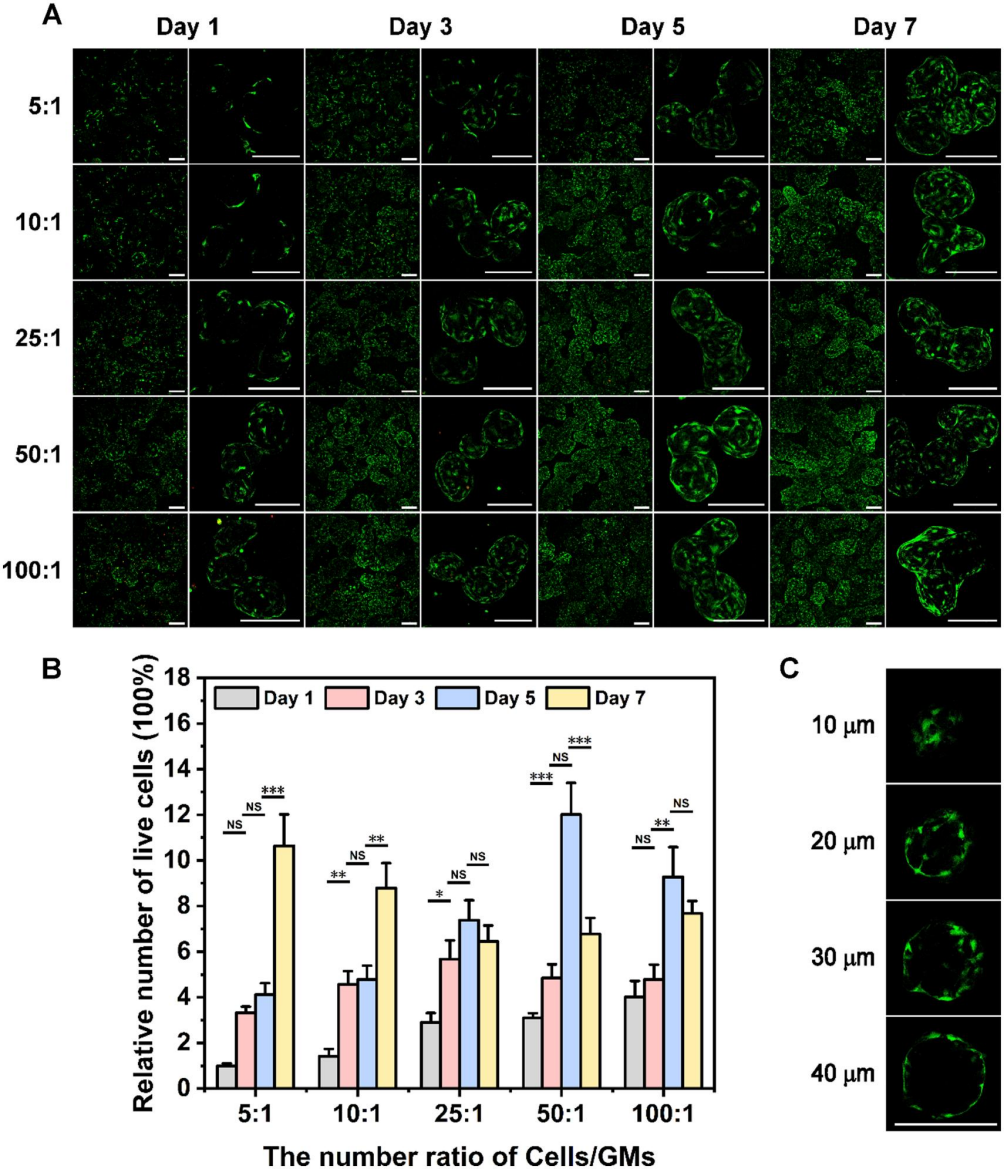
Cellular attachment, proliferation and migration in GMs were observed under live–dead staining, where green fluorescence (calcein-AM) and red fluorescence (PI) represent live and dead cells, respectively. (**A**) BMSCs were seeded in GMs at different ratios (100:1, 50:1, 25:1, 10:1 and 5:1) and cultured for 1, 3, 5 and 7 days (scale bar, 200 μm). (**B**) Relative number of BMSCs on GMs at gradient seeding ratios of 5:1, 10:1, 25:1, 50:1 and 100:1 after culture for 1, 3, 5 and 7 days determined by quantifying the fluorescence intensity of live–dead staining (*n* = 3, NS: not significant, *: *P *≤* *0.05, **: *P *≤* *0.01, ***: *P *≤* *0.001). (**C**) The cellular distribution and viability of BMSCs at different depths (10, 20, 30 and 40 μm) in GMs after culturing for 7 days (scale bar, 200 μm).

BMSCs and ATDC5 cells were respectively seeded into GMs at gradient number ratios of 5:1, 10:1, 25:1, 50:1 and 100:1 as described above. After 1, 3, 5 and 7 days of culture, cell activity and proliferation were observed via live–dead cell staining. As presented in Figure 2A, obvious green fluorescence of BMSCs could be observed on the GMs, and almost no dead cells were observed during culture. For ATDC5 cells, only a small amount of red fluorescence appeared with increasing culture time, which may be attributed to cellular contact inhibition caused by the high proliferation rate of ATDC5 cells (Figure S3A). Simultaneously, for both kinds of cartilage precursor cells, the low seeding ratio groups (5:1, 10:1 and 25:1) exhibited significant cell proliferation, as deduced from the increasing cell number with the extension of culture days (Figures 2B and S3B). In addition, after the cells were seeded on GMs for 7 days, they not only appeared on the surface of the GMs but also existed in the interior of the GMs (Figures 2C and S3C and Videos S1 and S2). SEM images further confirmed that the cells tightly attached to the surface of the GMs, including the surface of the pores (Figure S4). Collectively, BMSCs and ATDC5 cells maintained high activity, proliferation rates and migration capacities, which demonstrated that porous GMs were favorable for cell attachment, proliferation, and migration.

Notably, on Day 1, the number of cells (both BMSCs and ATDC5 cells) on GMs increased dramatically as the seeding number ratio increased. Then, the differences among groups with gradient ratios gradually decreased as the culture time increased, and there was little difference among the groups by Day 7. Furthermore, when the ratio was 100:1, the number of cells on each GM hardly increased with increasing incubation time. Therefore, it can be deduced that the number of cells each GM can carry is limited, and a seeding number ratio of 100:1 and an incubation time of 3 days were chosen for subsequent experiments to reduce culture and preparation time.

### Formation of cartilage microtissues

**Figure 3.**
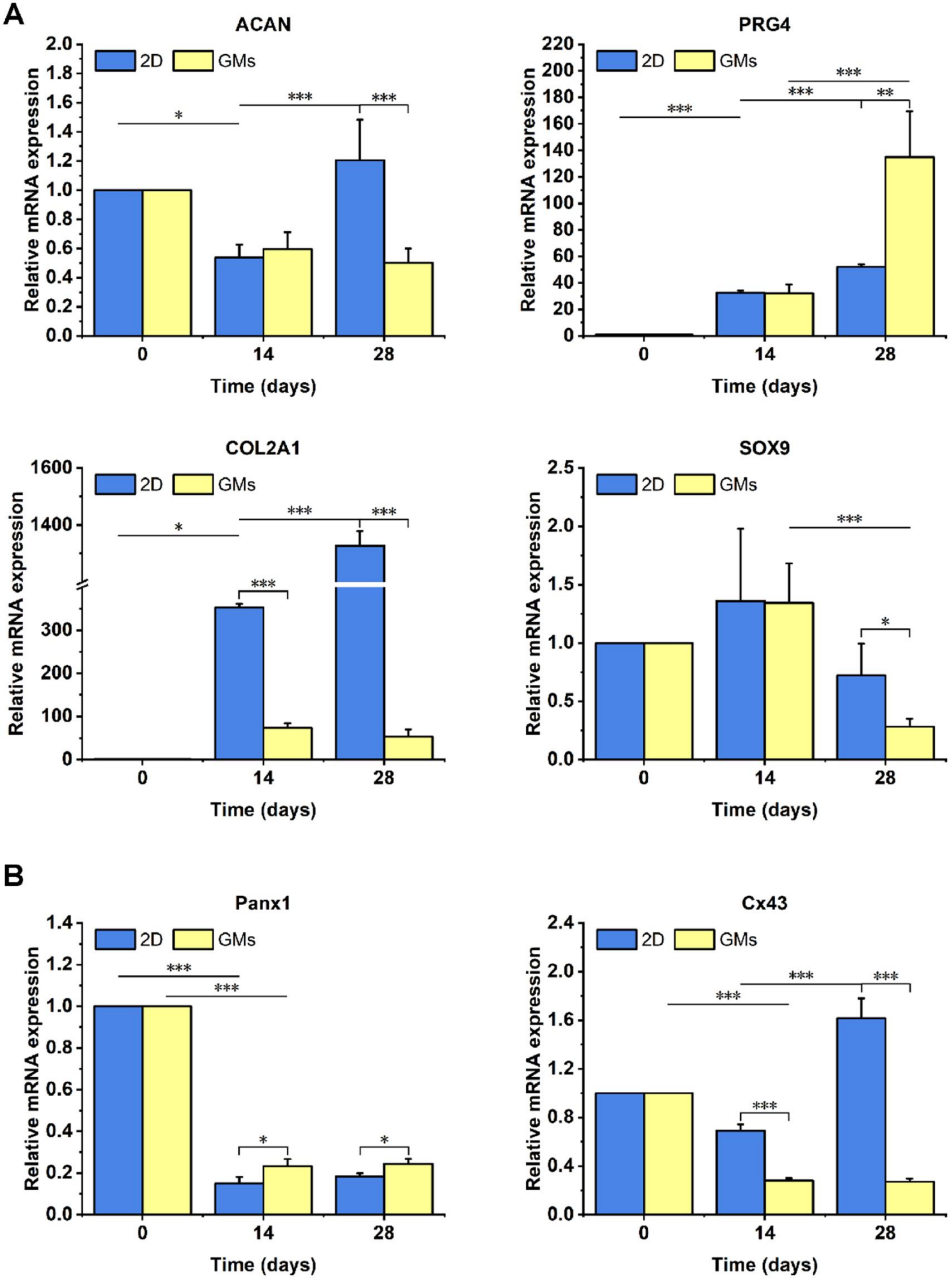
(**A**) Relative mRNA expression of chondrogenesis-related genes (*ACAN*, *PRG4*, *COL2A1* and *SOX9*) in BMSCs. (**B**) Relative mRNA expression of cell–cell connection-related markers (*Cx43* and *Panx1*) in BMSCs (*n* = 4, *: *P *≤* *0.05, **: *P *≤* *0.01, ***: *P *≤* *0.001).

BMSCs have been widely used in the regeneration of cartilage due to their superior self-renewal capacity, multidirectional differentiation ability and few ethical issues [57, 58]. In this work, BMSCs were employed to establish cartilage microtissues and further treat cartilage defects *in vivo*. After culture for 24 h, the BMSC-laden GMs were transferred and cultured in chondrogenic differentiation medium for 28 days. During this period, the mRNA expression levels of chondrogenesis-related markers, including *ACAN*, *PRG4*, *COL2A1* and *SOX9*, were determined by RT–qPCR analysis. As shown in Figure 3A, the expression of *ACAN* decreased first on Day 14 and subsequently stabilized in the GM group but increased slightly in the 2D group on Day 28. Notably, the mRNA expression of *PRG4* increased with prolonged incubation time, and the GM group significantly outperformed the 2D group on Day 28. In addition, the expression of *COL2A1* increased initially on Day 14 and remained stable on Day 28 in the GM group, while it continued to increase in the 2D group. These phenomena may be related to the extracellular microenvironment. Specifically, BMSCs laden in GMs were surrounded by gelatin with homology to collagen, so the relative expression of *COL2A1* was inhibited to some extent. Similar results were previously reported by Chang *et al.* [59]. The expression of *COL2A1* in MSCs cultured on gelatin scaffolds was significantly lower than that in MSCs cultured under 2D conditions. Meanwhile, the expression of *SOX9* increased initially, reached its highest level on Day 14 and then decreased gradually from Day 14 to Day 28, which was consistent with previous results. *SOX9*, a member of the Sry high-mobility-group box (SOX) gene family, was identified as the first transcription factor that directly activates a set of chondrocyte-specific ECM genes [60]. A previous study showed that the level of *SOX9* initially increased and then gradually decreased from Day 14 during chondrogenic formation [61]. Overall, the approximately 50-fold upregulation of *COL2A1* and 2-fold upregulation of *PRG4* on day 28 compared with those on day 0 cooperatively suggested the chondrogenic differentiation of BMSCs laden in the GMs.

RNA expression levels of cell–cell junction markers, including *Panx1* and *Cx43*, were also tested. It was previously reported that MSCs undergo a transition from primary cell–cell to cell–ECM interactions during the development of natural cartilage [62]. In other words, a reduction in cell–cell junctions initiates chondrogenesis in BMSCs. As illustrated in Figure 3B, the expression of *Cx43* in the GM group and that of *Panx1* in both groups decreased dramatically on Day 14 and then was maintained until Day 28. Meanwhile, the relative expression of *Cx43* in the 2D group decreased slightly on Day 14 but increased considerably on Day 28, which was significantly different from that in the GM group. In conclusion, the downregulation of cell–cell junction markers suggested that BMSCs in the GM group underwent a switch from stemness to differentiation.

**Figure 4.**
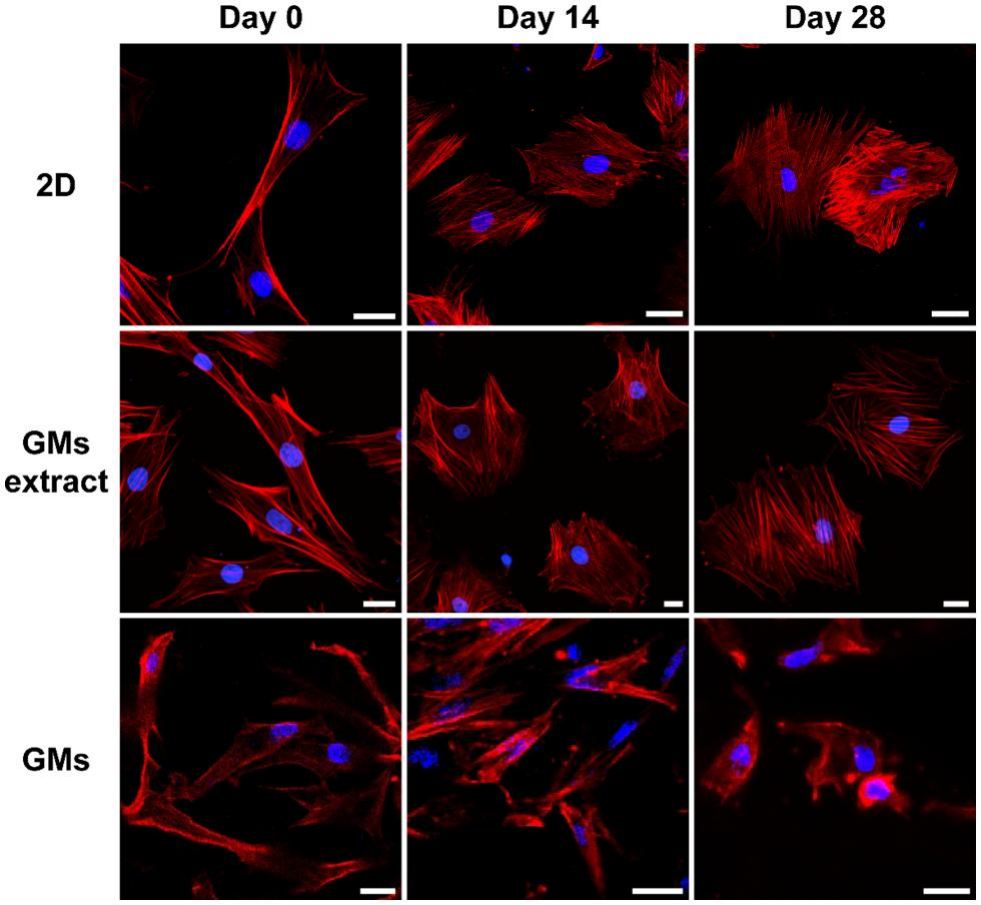
Images of cytoskeleton staining for BMSCs cultured in 2D plates without or with GM extract, and loaded on GMs after chondrogenic differentiation for 0, 14 and 28 days. The red (TRITC phalloidin) and blue (DAPI) fluorescence represent actin and nuclei, respectively (scale bar, 20 µm).

In addition, representative images of cytoskeleton staining showed the transformation of the phenotype throughout the incubation period. BMSCs transformed from a spindle to a round shape, gradually approaching the phenotype of chondrocytes (Figure 4). The phenotypes of cells cultured in 2D plates with or without GM extract were extremely similar, which excludes the effect of gelatin on the chondrogenic differentiation of cells.

**Figure 5.**
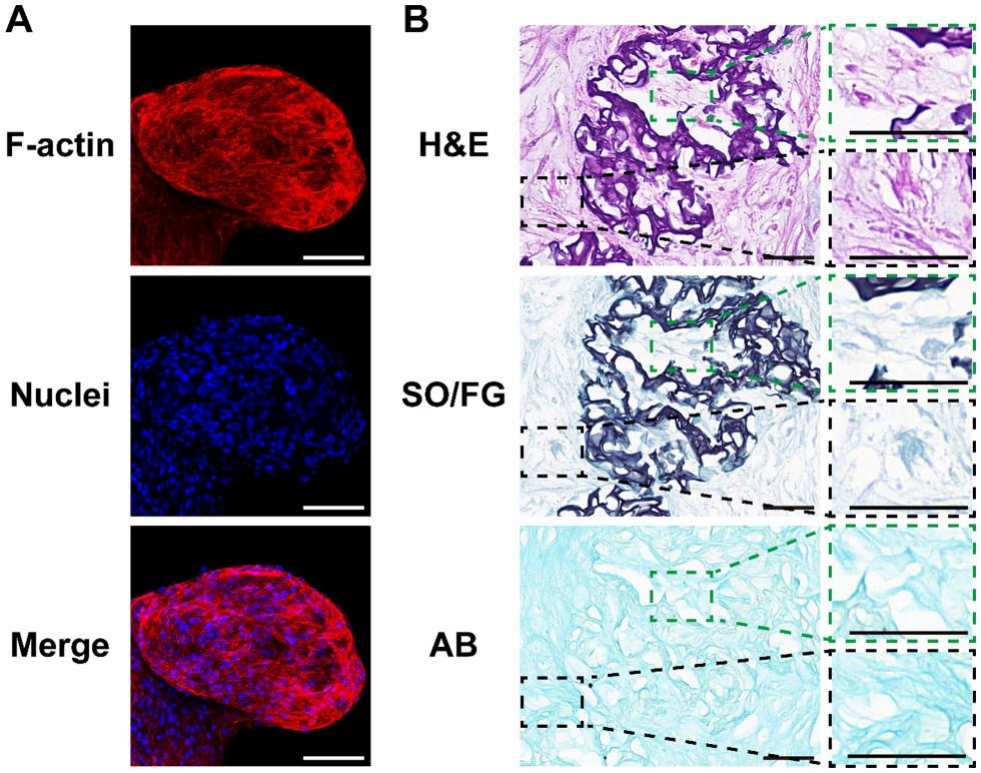
Observation of cell-laden GMs after chondrogenic differentiation for 28 days. (**A**) Cytoskeleton staining images of BMSC-laden GMs. The red (TRITC phalloidin) and blue (DAPI) fluorescence represent actin and nuclei, respectively (scale bar, 100 µm). (**B**) Histological analysis of BMSC-laden GMs, including H&E, so/FG and AB staining (scale bar, 40 µm). The staining images within and around the GMs were amplified in the dashed boxes, respectively.

Moreover, after chondrogenic differentiation for 28 days, cell-laden GMs were visualized via cytoskeleton and histological staining (Figure 5). As illustrated in Figure 5A, there were a considerable number of cells in the cell-laden GMs, which may have been connected by proliferated cells and produced ECM. Images of hematoxylin–eosin (H&E) staining are shown in Figure 5B, where the nuclei were stained violet blue with alkaline hematoxylin and the cytoplasm was stained pink with acid eosin. Many cells were observed both within and around the GMs. It is also worth noting that GMs were stained violet–blue. Most likely because the isoelectric point of gelatin is changed by acid proteoglycans secreted by cells, gelatin appears to be acidic and firmly bound to hematoxylin [63]. In addition, GMs were stained dark green by safranin O-fast green (SO/FG), while the rest were stained light green. In our opinion, FG bonds closely to gelatin in GMs and collagen in the remaining area of the microstructure. In addition, the entire microstructure was stained light blue by Alcian blue (AB), which indicated the deposition of acidic proteoglycans [64]. Collectively, cartilage-specific ECM (collagen and acidic proteoglycans) was secreted and deposited both in and around cell-laden GMs containing a mass of cells. Combined with the transformation of the cell phenotype, it can be deduced that cartilage microtissues were obtained after incubation with BMSC-laden GMs for 28 days.

ATDC5 cells were complementally employed to explore the effect of GMs on the chondrogenic differentiation of cells compared with 2D culture. The results on related gene expression (Figure S5) and phenotype transformation (Figure S6), together with a detailed discussion are provided in the Supplementary Material. In conclusion, GMs also play a positive role in the chondrogenic differentiation of ATDC5 cells.

### Efficacy of cartilage defect repair *in vivo*

**Figure 6.**
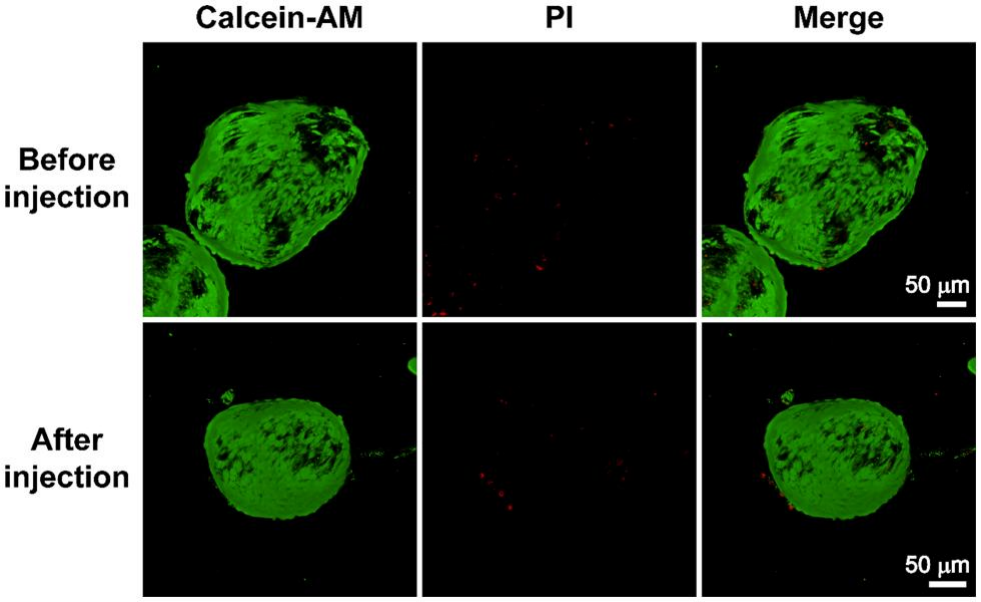
The viability of cells in cartilage microtissues before and after injection was observed under a laser-scanning confocal microscope after live–dead staining. The green (calcein-AM) and red (PI) fluorescence represent live cells and dead cells, respectively (scale bar, 50 µm).

The efficacy of the cartilage microtissues obtained above in repairing articular cartilage defects was evaluated *in vivo* by injecting them into the cartilage defect areas. First, the cell viability and integrity of the microtissues before and after injection were observed by live–dead cell staining. As illustrated in Figure 6, there were only a small number of dead cells both before and after injection. The lack of a significant increase in the number of dead cells indicates that cell viability was not significantly reduced by the shear force from the needle. Additionally, the integrity of the microtissues was well maintained.

**Figure 7.**
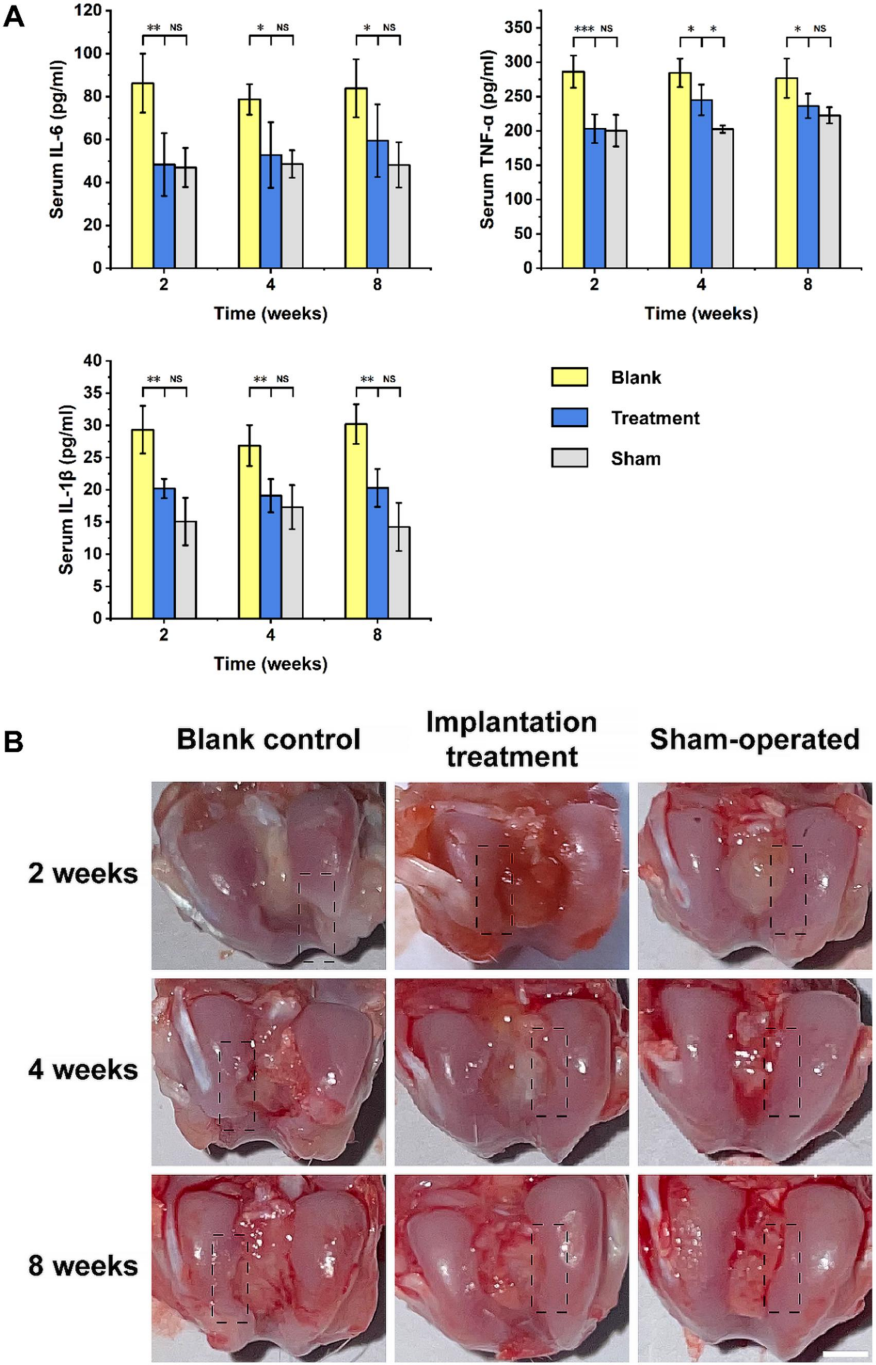
*In vivo* defect repair efficiency of cartilage microtissues. (**A**) The inflammatory response of rats was evaluated by detecting the secretion of inflammatory factors, including IL-1b, IL-6 and TNF-a (*n* = 6, NS: not significant, *: *P *≤* *0.05, **: *P *≤* *0.01, ***: *P *≤* *0.001). (**B**) Gross observation of the cartilage defect areas; the dashed boxes indicate the defect areas (scale bar, 2 mm).

The concentrations of inflammatory factors, including IL-6, TNF-α and IL-1β, in the blood of rats were quantified by ELISA. Previously, the effects of IL-6, TNF-α and IL-1β on articular cartilage injury and OA development have been well documented by several researchers [65–68]. In summary, they can disrupt microenvironmental homeostasis, inhibit anabolism, and amplify catabolism in cartilage. As shown in Figure 7A, the levels of IL-6, TNF-α and IL-1β in the treatment group were significantly lower than those in the blank control group at 2, 4 and 8 weeks after surgery. Moreover, there was no significant difference in the levels of inflammatory factors between the implantation treatment and sham-operated groups. Consequently, the implantation of microtissues can alleviate the inflammatory response, which is conducive to the repair of cartilage defects.

**Figure 8.**
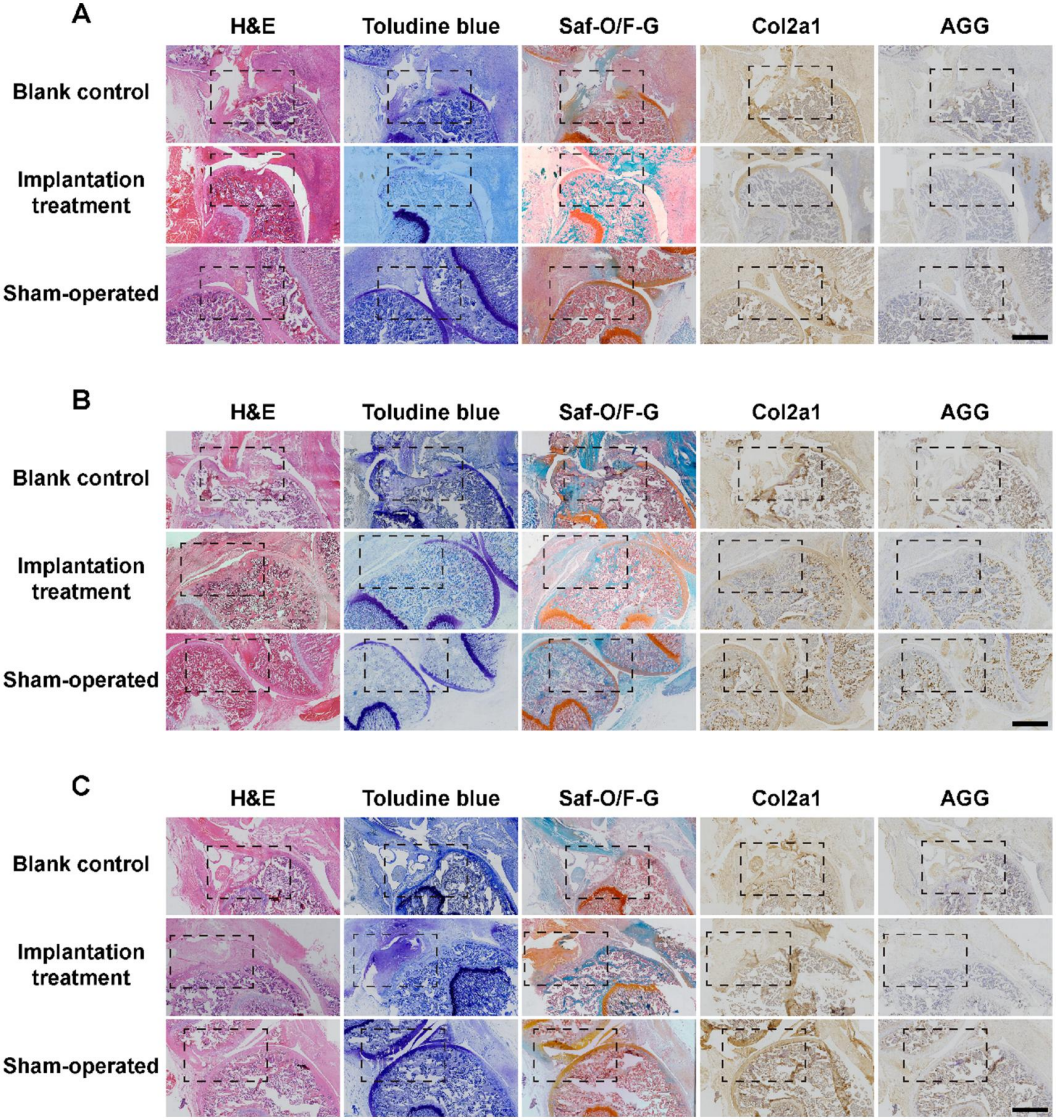
Histological analysis (H&E, toluidine blue staining, SO/FG) and IHC analysis of aggrecan and Col2a1 in cartilage defect areas at (**A**) 2 weeks, (**B**) 4 weeks and (**C**) 8 weeks postsurgery (the dashed boxes indicate the defect areas; scale bar, 2 mm).

Gross observation of cartilage defect areas at 2, 4 and 8 weeks postsurgery is presented in Figure 7B, in which the volume of defect areas in the implantation group was smaller than that in the blank control group. Furthermore, representative results of the histological analysis are shown in Figures 8 and S7. H&E staining images clearly showed that the defect areas in the implantation group were gradually filled with new tissues whose volume was larger than that of the counterpart of the blank control group. The toluidine blue and SO/FG staining results demonstrated the presence of acidic proteoglycans and new cartilage tissues in the original defect areas, respectively, the amounts of which were significantly distinct in the treatment group at 8 weeks postsurgery. In addition, IHC analysis of Col2a1 and aggrecan (AGG) revealed the secretion and deposition of collagen II and aggrecan, respectively, in new tissues. In summary, the defect areas in the treatment group were basically filled with new cartilage tissues, demonstrating the excellent treatment efficacy of cartilage microtissues.

## Conclusion

In this study, GMs were utilized to construct cartilage microtissues and subsequently repair articular cartilage defects. GMs are noncytotoxic and favorable for cell attachment, survival, proliferation and migration, as observed after coculturing BMSCs with GMs. Notably, cartilage microtissues were obtained through incubation of the BMSC-laden GMs in chondrogenic differentiation medium for 28 days. During the incubation period, the morphology of the BMSCs gradually became similar to that of the chondrocytes. The cells displayed upregulation of chondrogenesis-related markers and downregulation of cell–cell junction genes. The ability of GMs to promote the attachment, survival, proliferation, migration and differentiation of cartilage precursor cells was also verified using ATDC5 cells. The cartilage microtissues obtained from BMSC-laden GMs were further investigated by cytoskeleton staining and histological analysis, which revealed that cartilage-specific ECM components (collagen and acidic proteoglycans) were secreted and deposited. Animal studies have indicated that these injectable cartilage microtissues can effectively alleviate the inflammatory response and repair cartilage defects in rats. Prospectively, this may provide a promising method for minimally invasive cartilage defect treatment.

## Supplementary Material

rbae064_Supplementary_Data

## Data Availability

The data that support the findings of this study are available from the corresponding author upon reasonable request.
