# Supplementary material for: Injectable cartilage microtissues based on 3D culture using porous gelatin microcarriers for cartilage defect treatment
Source: Regen Biomater. 2024 Jun 4;11:rbae064. doi: 10.1093/rb/rbae064 (PMC11187498; doi:10.1093/rb/rbae064)
Supplement: rbae064_Supplementary_Data [file rbae064_supplementary_data.zip › Point by point response to the reviewers.docx]

**Point-by-point response to the Reviewers’ comments**

**Manuscript ID**: RB-2024-090

**Manuscript Title**: Injectable Cartilage Microtissues Based on 3D Culture Using Porous Gelatin Microcarriers for Cartilage Defect Treatment

**Author(s)**: Jing Zhu, Qiuchen Luo, Tiefeng Cao, Guang Yang *, Lin Xiao *

**Reviewer #1:**

Comments to the Author:

The authors developed injectable cartilage microtissues through the 3D culture of rat bone marrow mesenchymal stem cells (BMSCs) within porous gelatin microcarriers (GMs) and the induction of differentiation. The promotion effects of GMs on attachment, survival, proliferation, migration and differentiation of cartilage precursor cells were verified through the culture of BMSCs and ATDC5 cells. The in vivo animal experiment results on articular cartilage defect model demonstrated the cartilage repair efficacy of the BMSC-laden GMs. This is a meaningful study for developing injectable microtissues with minimum invasiveness for cartilage regeneration, and I would like to recommend its publication in Regenerative Biomaterials after the following issues are addressed.

1. To visualize the microstructure of the cell-laden microcarriers, the morphology characterization, such as scanning electron microscopy observation of the microcarriers seeded with BMSCs should be supplemented.

**Response:** We are grateful to the reviewer for this valuable suggestion. SEM images of the cell-laden GMs have been added to the revised manuscript.

**Revision:** Pages 7-8

**2.7. Morphology characterization of cell-laden GMs by SEM**

After washing with PBS, the cell-laden GMs were fixed in 2.5% glutaraldehyde for 2.5-3 h at room temperature, followed by dehydration with a graded ethanol series (30, 50, 70, 90, 95, and 100%) for 10 minutes in each solution. After air drying, they were carefully placed on conductive adhesive tape and then sputter-coated with gold for SEM observation.

Page 12: SEM images further confirmed that the cells tightly attached to the surface of the GMs, including the surface of the pores (**Fig. S4**).

Supplementary Material Page 6

**Fig. S4.** SEM images visualizing the microstructures of the ATDC5-laden GMs and the BMSC-laden GMs (scale bar, 20 μm).

2. For statistical analysis, all the levels of statistical significance appearing in figures should be involved in the captions of figures. Please check all the captions of figures and supplement the information.

**Response:** We thank the reviewer very much. We have checked all the figure captions and supplemented the missing information.

**Revision:** Page 13: the caption of Fig. 2.

(n=3, NS: not significant, *: *p* ≤ 0.05, **: *p* ≤ 0.01, ***: *p* ≤ 0.001)

3. In the last sentence on page 16, the authors claimed that 50-fold upregulation of COL2A1 on day 28 was observed in GMs. However, Figure 3A demonstrated an obvious downregulation of COL2A1 in GMs compared with that in 2D culture group on day 28, which should be explained.

**Response:** We apologize for the confusion. The mRNA expression of *COL2A1* on day 28 was compared with that on day 0, rather than comparing it between the GMs and the 2D culture group. The manuscript was revised to provide clearer expression. Additionally, the reason for the significant difference in COL2A1 between the GMs and the 2D culture group is explained in the manuscript on page 13: “These phenomena may be related to the extracellular microenvironment. Specifically, BMSCs laden in GMs were surrounded by gelatin with homology to collagen, so the relative expression of *COL2A1* was inhibited to some extent. Similar results were previously reported by Chang et al. The expression of *COL2A1* in MSCs cultured in gelatin scaffolds was significantly lower than that in MSCs cultured in 2D conditions.”

**Revision:** Pages 14-15: Overall, the approximately 50-fold upregulation of *COL2A1* and 2-fold upregulation of *PRG4* on day 28 compared with those on day 0 cooperatively suggested the chondrogenic differentiation of BMSCs laden in the GMs.

4. For in vivo animal experiment, how many experimental animals and knee joints were at each time point? Moreover, appropriate grading of histological results should be provided to demonstrate the treatment efficacy of cartilage microtissues.

**Response:** We are grateful to the reviewer for this valuable suggestion. There were six experimental animals and knee joints at each time point. We apologize for the inadequate expression, and an accurate description was revised in the manuscript. Additionally, appropriate grading of the histological results was also added to the revised manuscript.

**Revision:** Page 9: The rats were equally and randomly divided into the sham-operated group, the blank control group, and the implantation treatment group (n=6 for each group at each time point).

Page 10: For histological grading, sections corresponding to the center of each defect were selected and subjected to blind evaluation by 3 investigators. The histological grading scale (**Supplementary Table S2**) described by Wakitani et al. comprises 5 categories, the sum of which is scored from 0 (normal cartilage) to 14 (no repair tissue) [55,56].

Supplementary material Page 2

**Table S2.** Histological grading scale for cartilage defects

| **Category** |  | **Points** |
| --- | --- | --- |
| Cell morphology | Hyaline cartilage | 0 |
|  | Mostly hyaline cartilage | 1 |
|  | Mostly fibrocartilage | 2 |
|  | Mostly noncartilage | 3 |
|  | Noncartilaginous tissue | 4 |
| Matrix staining (metachromasia) | Normal (compared with host adjacent cartilage) | 0 |
|  | Slightly reduced | 1 |
|  | Significantly reduced | 2 |
|  | No metachromatic stain | 3 |
| Surface regularity | Smooth (>3/4^a^) | 0 |
|  | Moderate (1/2 < 3/4^a^) | 1 |
|  | Irregular (1/4 < 1/2^a^) | 2 |
|  | Severely irregular (<1/4^a^) | 3 |
| Thickness of the cartilage | 0 > 2/3^b^ | 0 |
|  | 1/3 < 2/3^b^ | 1 |
|  | <1/3^b^ | 2 |
| Integration of donor with host adjacent cartilage | Both edges integrated | 0 |
|  | One edge integrated | 1 |
|  | No integration | 2 |
| Total maximum | | 14 |

^a^ Total smooth area of the reparative cartilage compared with the whole area of the cartilage defect.

^b^ Average thickness of the reparative cartilage compared with that of the surrounding cartilage.

Page 20: Furthermore, representative results of the histological analysis are shown in **Fig. 8** and **Fig. S7**.

Supplementary material Page 9

**Fig. S7.** Comparison of the histological grading scores of regenerative tissues at 2 weeks, 4 weeks, and 8 weeks postsurgery. (n=3, NS: not significant, *: *p* ≤ 0.05, **: *p* ≤ 0.01, ***: *p* ≤ 0.001)

5. The microtissues were injected into the cartilage defect areas, I wonder if the integrity of the microtissues and the cell viability of BMSCs would deteriorate before and after the injection due to the shear force from the needle.

**Response:** This is a good question. We observed the cell viability and integrity of the microtissues before and after injection by live-dead cell staining, which has been added to the revised manuscript.

**Revision:** Pages 19-20: First, the cell viability and integrity of the microtissues before and after injection were observed by live-dead cell staining. As illustrated in **Fig. 6**, there were only a small number of dead cells both before and after injection. The lack of a significant increase in the number of dead cells indicates that cell viability was not significantly reduced by the shear force from the needle. Additionally, the integrity of the microtissues was well maintained.

Page 21

**Fig. 6.** The viability of cells in cartilage microtissues before and after injection was observed under a laser-scanning confocal microscope after live-dead staining. The green (calcein-AM) and red (PI) fluorescence represent live cells and dead cells, respectively (scale bar, 50 µm).

6. Gross observation images shown in Figure 6 are not clear enough to evaluate the cartilage defect repair, and the authors should update images with higher quality and higher resolution.

**Response:** We thank the reviewer very much for this suggestion. Images with higher resolution were added to the manuscript. We apologize for the insufficient quality. We photographed the whole femur during the experiment, and those images are partial magnifications of the corresponding whole bones. We will pay more attention to and correct this problem in the future.

**Revision:** Page 22

**Fig. 7.** *In vivo* defect repair efficiency of cartilage microtissues. (A) The inflammatory response of rats was evaluated by detecting the secretion of inflammatory factors, including IL-1b, IL-6, and TNF-a (n=6, NS: not significant, *: *p* ≤ 0.05, **: *p* ≤ 0.01, ***: *p* ≤ 0.001). (B) Gross observation of the cartilage defect areas; the dashed boxes indicate the defect areas (scale bar, 2 mm).

**Reviewer #2:**

Comments to the Author:

Current study explored a typical tissue-engineering approach to regenerate cartilage defects with cell-loaded gelatin microcarriers. It did acquire positive results from routine experiment, but contributed little to the ground. It is difficult to achieve novelty with well-used mesenchymal stem cells, commercialized gelatin microcarriers as well as not-new combination methodology.

Frankly, I was trained as a tissue engineer two dozen years ago when tissue engineering witnessed its bubble and peak. However, we have to adjust when looking back and asking us that this strategy can provide “all-in-one” formula for all tissue. The answer is definitely not. We have to move in to explore with novel strategy and perspectives.

**Response:** We are grateful for the reviewer's insightful comments and appreciate the opportunity to reflect on the broader impact and novelty of our work. We acknowledge that the use of mesenchymal stem cells and gelatin microcarriers, along with the methodology employed, may not represent groundbreaking innovations in the field of tissue engineering. However, we believe that the specific application and integration of these established components in our study hold distinct value.

Our research marks, to our knowledge, the first instance of utilizing gelatin microcarriers (GMs) for the construction of in vitro cartilage microtissue, followed by their direct application in vivo for cartilage repair through an injectable approach. This method not only demonstrates a practical application but also emphasizes a minimally invasive strategy for cartilage repair, which is significant given the clinical challenges in treating cartilage defects efficiently and effectively.

Moreover, the compatibility of the GMs' composition and microstructure with the requirements for cartilage tissue engineering is particularly noteworthy. This synergy enhances the potential for successful tissue integration and regeneration, offering a promising avenue for future clinical applications.

We recognize the need for novel strategies and perspectives, as highlighted by the reviewer. In response, our ongoing and future work is directed towards innovating within the frameworks of established methodologies, striving to enhance their applicability and effectiveness in clinical settings. We are committed to advancing the field by exploring new materials, scaffold designs, and biointeractive signals that could redefine the standards of tissue engineering practices.

In conclusion, while our current approach builds on existing technologies, its unique application in the context of cartilage repair represents a meaningful step forward in the field. We appreciate the reviewer's call for innovation and are inspired to further our research with novel contributions that align with the evolving needs of tissue engineering.

**Reviewer #3:**

Comments to the Author:

The author introduced a BMSCs-laden GM for cartilage regeneration and conducted the in vivo and in vitro evaluation. However, a major revision is necessary to complete the manuscript. My comments are as follows:

1. The physicochemical performance of GM is a factor influencing the biological property. Therefore, the physicochemical properties of GM should be supplemented, including porosity, pore size, and so on.

**Response:** We are grateful to the reviewer for this valuable suggestion. The physicochemical performance of the GM was supplemented in the manuscript.

**Revision:** Page 6

**2.3. Characterization of GMs**

The morphology of the GMs was monitored by scanning electron microscopy (SEM) at an accelerating voltage of 20.0 kV. A Fourier transform infrared (FTIR) spectrophotometer was used to characterize the chemical composition of the GMs. Spectral measurements were performed within the mid-infrared range of 4000-500 cm^-1^. All measurements were performed in a dry atmosphere at room temperature, and the background spectrum was obtained from a pure KBr pellet.

Page 11: The 3D TableTrix® gelatin microcarriers (GMs) used in this study have interconnected macroporous structures with a porosity of 95.73% ± 0.28%, pore sizes in the range of 30-80 μm, and a size distribution ranging from 150 μm to 300 μm [36,37]. Their morphology was visualized by SEM (**Fig. S1**A). Their chemical composition was characterized by FTIR (**Fig. S1**B). Five major peaks, i.e., at 3301.4 cm^-1^, 1654.8 cm^-1^, 1535.9 and 1450.4 cm^-1^, as well as 1229.5 cm^-1^ correspond to the amide A, amide Ⅰ, amide Ⅱ, and amide Ⅲ regions of gelatin, respectively [57].

Supplementary Material Page 3

**Fig. S1.** Physicochemical characterization of 3D TableTrix® GMs. (A) SEM image of the GMs (scale bar, 50 μm). (B) FTIR spectrum of the GMs.

2. BMSCs were finally selected for the in vivo evaluation. What is the reason for the choose of ATDC5 cells for cell viability and proliferation evaluation?

**Response:** We thank the reviewer very much for this important question. ATDC5 cells are normally used for *in vitro* studies in cartilage tissue engineering. Specifically, ATDC5, a mouse cartilage precursor cell line derived from teratoma cells, has been widely used to explore biochemical and developmental mechanisms throughout the chondrogenic differentiation process. Therefore, in this study, ATDC5 cells were utilized to complementally verify that GMs are favorable for the attachment, survival, proliferation, migration and differentiation of cartilage precursor cells, which supported the studies and results using BMSCs.

3. To exclude the effect of gelatin on cells and explain the advantages of GM, the medium containing equivalent gelatin solution should be employed in 2D culture in Figure 4.

**Response:** We express our sincere gratitude to the reviewer for the insightful suggestion. To exclude the effect of gelatin on cells and explain the advantages of GM, images of cytoskeleton staining for BMSCs cultured in 2D plates supplemented with GM extract have been added to the revised manuscript and discussed.

**Revision:** Page 9: Additionally, to exclude the effect of gelatin composition on cells, the phenotype of BMSCs cultured in 2D plates supplemented with GM extract was also observed. GM extract was obtained by immersing GMs in cell culture medium at the same concentration as that used for landing BMSCs on GMs. After extraction for 72 h, the extract was collected by centrifugation to remove GMs.

Page 15: The phenotypes of cells cultured in 2D plates with or without GM extract were extremely similar, which excludes the effect of gelatin on the chondrogenic differentiation of cells.

Page 18

**Fig. 4.** Images of cytoskeleton staining for BMSCs cultured in 2D plates without or with GM extract, and loaded on GMs after chondrogenic differentiation for 0, 14, and 28 days. The red (TRITC phalloidin) and blue (DAPI) fluorescence represent actin and nuclei, respectively (scale bar, 20 μm).

4. One Way Anova was not feasible in Figure 3.

**Response:** We thank the reviewer very much for the helpful suggestion. This has been revised in the manuscript.

**Revision:** Page 11: The statistical significance of differences between two groups was determined by a two-tailed independent-samples t test. The statistical significance of differences among multiple groups was determined via one-way analysis of variance (ANOVA) using the Tukey post hoc test.

5. The modeling of animal experiments requires clarification.

**Response:** We sincerely appreciate the helpful suggestion. A clearer description of the animal experiments has been provided in the revised manuscript.

**Revision:** Pages 9-10

Fifty-four SD rats (6 weeks old, body weight: 260-280 g) were used to create knee cartilage defect models to evaluate the repair efficiency of the microtissues. The rats were equally and randomly divided into a sham-operated group, a blank control group, and an implantation treatment group (n=6 for each group at each time point). Following general anesthesia, a longitudinal skin incision (length: 2 cm) was created on the surface of the right knee joint. The knee joint was opened through a medial parapatellar approach. The patella was dislocated laterally to expose the femoropatellar groove. Cartilage defects (diameter: 3 mm, height: 1.5 mm) were subsequently constructed on the patellar groove by using a 3 mm diameter hand drill, after which cartilage microtissues from BMSC-laden GMs were implanted into the defects. In addition, the cartilage defects of the rats in the blank control group were not treated, while those in the sham-operated group were not constructed. After the surgery, the animals were returned to their cages without joint immobilization.

6. The AB staining in Figure 5 was not persuasive. The significant differences were difficult to distinguish from histological staining results for Blank group and treatment group. The semi-quantitative analysis and high power field for staining results was necessary.

**Response:** We appreciate the reviewer’s helpful suggestion. Figure 5 shows that after chondrogenic differentiation for 28 days, cell-laden GMs were visualized via cytoskeleton and histological staining. In Figure 5B, the green dashed box and black dashed box amplified the staining images within and around the GMs, respectively, rather than within the blank group and treatment group. We apologize for the confusion caused by inadequate expression, and precise descriptions have been provided in the revised manuscript.

**Revision:** Page 19

**Fig. 5.** Observation of cell-laden GMs after chondrogenic differentiation for 28 days. (A) Cytoskeleton staining images of BMSC-laden GMs. The red (TRITC phalloidin) and blue (DAPI) fluorescence represent actin and nuclei, respectively (scale bar, 100 μm). (B) Histological analysis of BMSC-laden GMs, including H&E, SO/FG, and AB staining (scale bar, 40 μm). The staining images within (green dashed box) and around (black dashed box) the GMs were amplified.
